# Molecular Epidemiology of Carbapenem-Resistant *Klebsiella pneumoniae* in a Tertiary Hospital in Northern China

**DOI:** 10.1155/2022/2615753

**Published:** 2022-12-03

**Authors:** Shuqing Wang, Huanhuan Dong, Meiqi Wang, Wenbo Ma, Yue Cheng, Junliang Zhou, Yongming Cheng, Hui Xu, Xiaochen Yu

**Affiliations:** ^1^Department of Clinical Laboratory, Harbin Medical University Cancer Hospital, Harbin 150081, China; ^2^Laboratory Diagnosis Department of the First Affiliated Hospital of Harbin Medical University, Harbin 150007, China

## Abstract

**Background:**

In recent years, carbapenem-resistant* Klebsiella pneumoniae* (CRKP) has emerged rapidly in China with the abuse and overuse of antibiotics, and infections caused by CRKP pose a serious threat to global public health safety. The present study aimed to explore the epidemiological characteristics of CRKP isolates in Northern China and to elucidate their drug resistance mechanisms.

**Methods:**

45 CRKP strains were consecutively collected at a teaching hospital from March 1st, 2018 to June 30th, 2018. Antimicrobial susceptibility was determined by the VITEK2 compact system and microbroth dilution method. Polymerase chain reaction (PCR) and sequencing were used to analyze multilocus sequence typing (MLST), drug resistance determinants, and plasmid types. The transfer of resistance genes was determined by conjugation. All statistical analysis was performed using SPSS 22.0 software.

**Results:**

All 45 isolates showed multidrug resistance (MDR). MLST analysis showed ST11 (48.9%, 22/45) was the most frequent type. All of the 45 CRKP isolates contained carbapenemase genes, extended-spectrum* β*-lactamase (ESBL) genes, and plasmid-mediated quinolone resistance (PMQR) genes. For carbapenemase genes, KPC-2 (93.3%, 42/45) was the main genotype, and followed by GES (37.8%, 17/45) and NDM-1 (11.1%, 5/45). Plasmid typing analysis showed that IncFII and IncFIB were the most prevalent plasmids. The carbapenem resistance rate of *K.pneumoniae* was 11.4% and ICU was the main CRKP infection source.

**Conclusions:**

ST11 is the most frequent sequence type and KPC-2 is the predominant carbapenemase of CRKP strains in Northern China. KPC-2-ST11 are representative clonal lineages.

## 1. Introduction

*Klebsiella pneumoniae*, a common opportunistic pathogen, can cause severe pneumonia, bloodstream infection, urinary tract infection, wound or surgical site infection, and meningitis, especially, in immunocompromised patients [1], which accounts for approximately 10% of hospital-acquired infections [2]. In recent years, with the abuse and overuse of antibiotics, particularly the third-generation cephalosporins and carbapenems, carbapenem-resistant *Klebsiella pneumoniae* (CRKP) has emerged rapidly in China, with the carbapenem resistant rate increasing from 3% in 2005 to 25.5% in 2021 based on the data of CHINET [3, 4]. The majority of CRKP isolates was resistant to more than three kinds of antibiotics and was defined as multidrug resistance (MDR) [5]. Therefore, infections caused by CRKP pose serious threats to global public health security and an enormous challenge to antimicrobial therapy.

The major carbapenem resistance mechanisms of CRKP are the production of enzymes capable of hydrolyzing carbapenems, including class *A β*-lactamases (KPC, GES), class *B β*-lactamases (IMP, NDM, and VIM) and Class *D β*-lactamases (OXA-48) [6], with KPC and NDM being the predominant enzymes in China [7]. Other resistance mechanisms include overexpression of efflux pump genes [8], porins protein mutation and producing AmpC, and ESBLs simultaneously [9, 10].

The spread of CRKP is rapidly occurring in hospital-acquired infections, as the emergence of mobile gene elements carrying the carbapenem-resistance gene can shuttle easily through horizontal or vertical gene transfer in the same or different species, especially, result in accelerated growth of resistant strains across many species of enterobacterial species [11]. Therefore, studying the molecular epidemiology of CRKP was necessary to limit the spread of antimicrobial resistance and can provide an empirical treatment strategy for the clinicians.

In this study, we investigated the molecular characteristics, plasmid typing, and antimicrobial resistance gene of carbapenem-resistant *Klebsiella pneumoniae* (CRKP) isolates from a tertiary hospital in Northern China, so as to provide a basis for the control and prevention of nosocomial infections.

## 2. Materials and Methods

### 2.1. Study Design and Setting

We performed a retrospective study from March 1st, 2018 to June 30th, 2018 in the First Affiliated Hospital of Harbin Medical University, a 6498-bed tertiary hospital in Northern China. We reviewed the microbiology laboratory database of any patients with CRKP infection during this period. Only the first positive culture of CRKP for each patient was included in this study, and patients with insufficient medical records were excluded from this study. This study was approved by the ethics committee of the First Affiliated Hospital of Harbin Medical University.

### 2.2. Bacterial Strains

In this study, a total of 395 strains of non-repetitive *K. pneumoniae* were collected consecutively from March 1st, 2018 to June 30th, 2018 in the First Affiliated Hospital of Harbin Medical University. These strains come from different clinical samples, including sputum, blood, pus, ascites, cerebrospinal fluid, bile, and secretions, among which 45 strains were identified as carbapenem-resistant *K. pneumoniae*. The CRKP strains were defined as resistant to at least one of carbapenem antibiotic (imipenem, meropenem, or ertapenem). The clinical information was obtained from the medical record. Identification of the *K*. *pneumoniae* isolates was performed by the VITEK2 compact system (BioMérieux, Marcy l'Etoile, France) and the matrix-assisted laser desorption ionization-time of flight-mass spectrometry (MALDI-TOF-MS) (BioMérieux, Marcy l'Etoile, France). The *Escherichia coli* (ATCC25922, ATCC8739), *Pseudomonas aeruginosa* (ATCC27853) were used as reference strains. The rifampin-resistant strain *E. coli* EC600 was used as the recipient strain for conjugation experiments.

### 2.3. Antimicrobial Susceptibility Testing

The antimicrobial susceptibility test was performed by using the VITEK2 compact system (BioMérieux, Marcy l'Etoile, France), antibiotics used included piperacillin/tazobactam (TZP), amoxicillin/clavulanic acid (AMC), cefepime (FEP), cefazolin (CFZ), ceftriaxone (CRO), cefoperazone-sulbactam (SCF), cefoxitin (FOX), imipenem (IPM), meropenem (MEM), ertapenem (ETP), gentamicin (GM), amikacin (AMK), cztreonam (ATM), tobramycin (TOB), ciprofloxacin (CIP), levofloxacin (LEV), trimethoprim/sulfamethoxazole (SXT), tigecycline (TGC), and colistin (COL). The CRKP MICs of colistin, imipenem, and rifampin were further tested by the broth microdilution method, and the results were interpreted according to the Clinical and Laboratory Standards Institute (CLSI, 2018) guidelines. The breakpoint of tigecycline was based on the US Food and Drug Administration standard (FDA).

### 2.4. Multilocus Sequence Typing (MLST)

The MLST analysis was conducted by amplifying seven housekeeping genes including *gapA, infB, mdh, pgi, phoE, rpoB, and tonB* for the genetic relatedness of 45 CRKP isolates. The sequence types (STs) of each strain were sequenced and blasted using the online database at https://bigsdb.pasteur.fr/klebsiella/klebsiella.html. The phylogenetic tree was conducted by BioNumerics software with the maximum likelihood method, and a minimum spanning tree was also generated by BioNumerics software.

### 2.5. Antimicrobial Resistance Genes Detection

PCR was used to detect the carbapenemase genes (*bla*_KPC-2_, *bla*_IMP_, *bla*_VIM_, *bla*_NDM-1_, *bla*_GES_, and *bla*_OXA-48_), ESBL genes (*bla*_SHV-11_, *bla*_SHV-12_*, bla*_SHV-33_*, bla*_SHV-27,_*bla*_SHV-28,_*bla*_TEM-1_, *bla*_OXA-1_, *bla*_CTX-M-1_, *bla*_CTX-M-2_, *bla*_CTX-M-3,_*bla*_CTX-M-9_, and *bla*_CTX-M-15_), AmpC genes (*bla*_MOX_, *bla*_CIT_, *bla*_DHA_, *bla*_ACC_, *bla*_EBC_, and *bla*_FOX_), aminoglycoside resistance determinants (ARD) (*aac (6′)-Ib, aac (3′)-IIa, aac (3′)-Ia, aac (3′)-IV, aph (3′)-VI, ant (2′)-Ia, ant (3′)-I*) and plasmid-mediatedquinolone-resistant (PMQR) genes (*qnrA*, *qnrB*, *qnrC*, *qnrS*, *qepA*, and *oqxA*). The primers were used as described previously [12–18]. All positive PCR products were confirmed by sequencing and compared with those found in the GenBank nonredundant DNA database using the BLAST algorithm available at NCBI (https://blast.ncbi.nlm.nih.gov/).

### 2.6. Conjugation Experiment

The conjugation experiments were carried out with the method described previously with slight modifications [19]. In brief, the rifampin-resistant* E. coli* EC600 was used as the recipient, and the 45 CRKP isolates were used as donors. The isolates were grown on blood agar plates at 37°C overnight. Each of the donor and recipient bacteria were grown in 3 ml of LB broth at 37°C for 4 h. The mixture of the donor and recipient isolates at a volume ratio of 3 : 1 was spotted on a 1 cm^2^ hydrophilic nylon membrane filter with a 0.22 *μ*m pore size that was placed on an blood agar plate and then incubated for mating at 37°C for 24 h. Transformants were selected on LB agar containing imipenem (0.5 *μ*g/ml) and rifampin (100 *μ*g/ml).

### 2.7. Plasmid Replicons Analysis

PCR-based replicon typing was performed for the determination of the following plasmid types: IncFII, IncFIIK, IncFIA, IncFIB, IncHII, IncII, IncX, IncA/C, IncL/M, IncB/O, and CoIE, and the primers were as described by Johnson et al. and Potron et al. [20, 21]. The DNA of each isolate was extracted by the boiling method. All PCR amplifications, except the IncFII, were performed with the following amplification scheme: initial denaturation of 5 min at 94°C, followed by 30 cycles of denaturation (94°C, 60 s), annealing (60°C, 30 s), extension (72°C, 60 s), and with a final extension of 5 min at 72°C. The IncFII amplification was performed with an annealing temperature of 52°C. The PCR products were sequenced and blasted in PubMed (https://blast.ncbi.nlm.nih.gov/) to confirm the plasmid types.

### 2.8. Statistical Analysis

All statistical analysis was performed using SPSS 22.0 software. We used the chi-square test or Fisher's exact test to analyze the categorical variables and the Student's *t*-test or ANOVA to analyze the continuous variables. *P* < 0.05 was considered statistically significant.

## 3. Results

### 3.1. Clinical and Demographic Characteristics

A total of 395 nonrepeated *K*. *pneumoniae* isolates were collected in the First Affiliated Hospital of Harbin Medical University from March 1st 2018 to June 30th 2018, 45 of which were identified as carbapenem-resistant* K*. *pneumoniae*. The clinical and demographic characteristics of CRKP patients were summarized in Table 1. The patients' ages ranged from 1 to 93 years old with a median age of 53 years old, and twenty-seven patients (60%, 27/45) were male. From their medical records, patients with CRKP infection were mainly from intensive care unit (ICU) (48.9%, 22/45), and hepatobiliary surgery (22.2%, 10/45). The most common specimen sources were sputum (40%, 18/45) and blood (35.6%, 16/45), which accounted for 75.6% of all cases. The 45 CRKP isolates were mainly isolated from patients with brain diseases (31.1%, 14/45), followed by pancreatic diseases (15.6%, 7/45), blood diseases (11.1%, 5/45), biliary diseases (11.1%, 5/45), and abdominal pain (11.1%, 5/45).

### 3.2. Antimicrobial Susceptibility Testing

**Figure 1:**
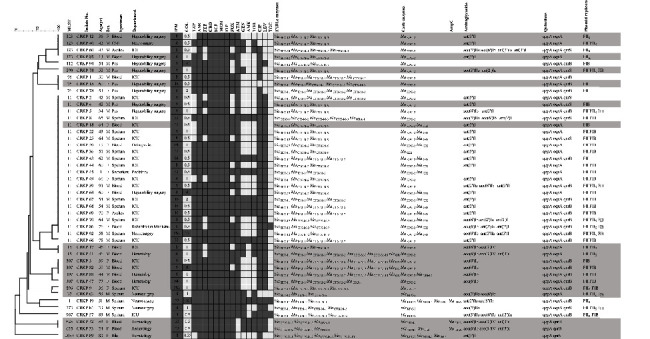
Dendrogram generated from adobe illustrator CS6 showing the multilocus sequence typing (MLST) of 45 CRKP isolates together with their ward, antimicrobial susceptibility profiles, resistant genes, and replicon types.

The antimicrobial resistance profiles of 45 CRKP isolates against 20 common antibiotics were shown in Table 2. The drug resistance rates of ICU patients infected with CRKP to aztreonam, ciprofloxacin, and levofloxacin were higher than those of non-ICU patients, while the drug resistance rates of ICU patients infected with CRKP to cefoxitin and colistin were lower, and the differences were statistically significant (*P* < 0.05). In addition, MICs of imipenem were tested by broth microdilution and all of the 45 isolates were resistant to imipenem with the MIC_50_ (8 *μ*g/ml) and MIC_90_ (64 *μ*g/ml), respectively. The MIC distribution of imipenem was shown in Figure 1. Notably, all of the CRKP isolates were MDR. The resistance rates to carbapenems, cephalosporins, and fluoroquinolones were relatively high, with the resistance rates more than 70%, and the resistance rates to tigecycline and colistin were low, with the resistance rates about 10%.

### 3.3. Molecular Typing

**Figure 2:**
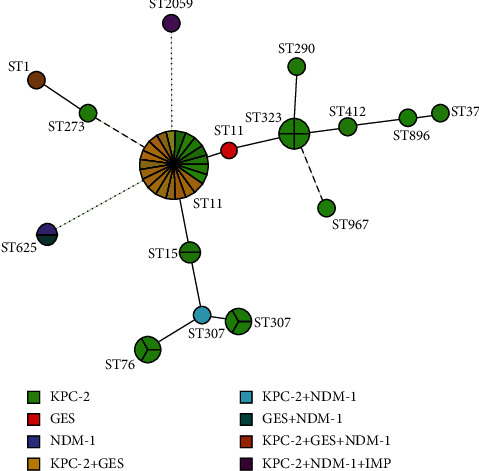
The distribution of carbapenemase genes among different STs. Minimum spanning trees of 45 CRKP strains. Each node represents a single ST. The size of the nodes was proportional to the number of isolates in the representative ST.

Among the 45 CRKP isolates, 14 sequence types (ST1, ST11, ST15, ST37, ST76, ST273, ST290, ST307, ST323, ST412, ST625, ST869, ST967, and ST2059) were identified in this study (Figure 1). ST11 was the most common clone, accounting for 48.9% of the 45 CRKP isolates, followed by ST307 (8.9%, 4/45), ST323 (8.9%, 4/45), ST76 (6.7%, 3/45), ST15 (4.4%, 2/45), ST625 (4.4%, 2/45), ST1 (2.2%, 1/45), ST37 (2.2%, 1/45), ST273 (2.2%, 1/45), ST290 (2.2%, 1/45), ST412 (2.2%, 1/45), ST896 (2.2%, 1/45), ST967 (2.2%, 1/45), and ST2059 (2.2%, 1/45). CRKP isolates that co-produced* bla*_KPC-2_ and *bla*_GES_ all belonged to ST11, whereas the strains carrying *bla*_NDM-1_ belonged to different STs, including ST307, ST625, ST1, and ST2059. The distribution of carbapenemase genes in different STs was shown in Figure 2. Compared with other STs, the resistance rates of ST11 to amikacin, aztreonam, ciprofloxacin, and levofloxacin were higher while to piperacillin/tazobactam, cefoxitin, gentamicin, and trimethoprim/sulfamethoxazole were lower, and the difference was statistically significant (*P* < 0.05). The distributions of antibiotic resistance rates for ST11 and other STs were shown in Table 2. The MIC_50_ and MIC_90_ of imipenem for ST11 in CRKP were 8 and 64 *μ*g/ml, respectively. Imipenem MICs of different STs of 45 CRKP isolates were shown in Table 3.

### 3.4. Resistance Genes Detection

All 45 CRKP isolates were found to carry carbapenemase genes, among which *bla*_KPC-2_ (93.3%, 42/45) was commonly detected, followed by *bla*_GES_ (37.8%, 17/45), *bla*_NDM-1_ (11.1%, 5/45), and *bla*_IMP_ (2.2%, 1/45), while *bla*_OXA-48_ and *bla*_VIM_ genes were not detected. Isolates often carry one, occasionally two or three different types of carbapenemase genes. CRKP isolates that coproduced *bla*_KPC-2_ and *bla*_GES_ had a higher MIC_50_ and MIC_90_ compared to other carbapenemase-producing CRKP. All carbapenemase genes were accompanied by ESBL and PMQR genes, 86.7% (39/45) of CRKP isolates harbored aminoglycoside resistance genes and only 3 strains harbored AmpC group genes. The distribution of imipenem MIC in different resistance gene types was shown in Table 4.

Of the 45 CRKP isolates, all isolates carried the *bla*_CTX-M_ group gene (*n* = 45; 100%), 91.1% (*n* = 41) carried *bla*_SHV_ group gene, 44.4% (*n* = 20) carried *bla*_TEM-1_, and 17.8% (*n* = 8) carried *bla*_OXA-1._ In the *bla*_CTX-M_ group, *bla*_CTX-M-2_ was the most prevalent genotype (44/45, 97.8%), followed by *bla*_CTX-M-3_ (20/45, 44.4%), *bla*_CTX-M-1_ (18/45, 40%), *bla*_CTX-M-9_ (17/45, 37.8%), and *bla*_CTX-M-15_ (5/45, 11.1%). In *bla*_SHV_ group, *bla*_SHV-11_ was the most prevalent genotype (21/45, 46.7%), followed by *bla*_SHV-12_ (15/45, 33.3%), *bla*_SHV-33_ (2/45, 4.4%), *bla*_SHV-27_ (2/45, 4.4%), and *bla*_SHV-28_ was detected in only one strain. In AmpC enzymes genes, only *bla*_DHA_ was detected in 3 strains. A complete description of the resistant genes characterized in the 45 CRKP isolates was available in Figure 1.

### 3.5. Resistance Genes Transfer and Plasmid Incompatibilities

The carbapenemase genes of eighteen CRKP strains were successfully transferred in the conjugation experiment. The antimicrobial MICs of the 18 CRKP donors and *E. coli* EC600 transconjugants were presented in Table 5. In general, the resistance profiles to carbapenems and cephalosporins of the eighteen transconjugants were similar to those of the CRKP donor strains, demonstrating the transfer of resistance genes.

**Figure 3:**
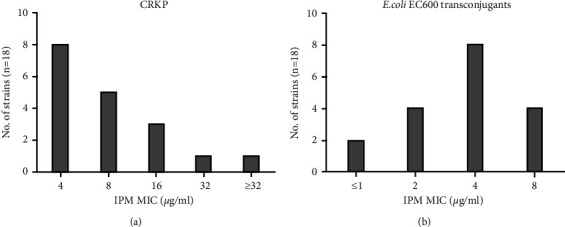
The distribution of MIC values of imipenem antibiotic determined by broth microdilution method. (a) MICs of IPM against CRKP. (b) MICs of IPM against *E.coli* EC600 transconjugants.

Among which, *β*-lactamase genes including *bla*_KPC-2_, *bla*_NDM-1_, *bla*_TEM-1_, *bla*_CTX-M-15_, *bla*_CTX-M-9_, and *bla*_*OXA-1*_ were successfully transferred by conjugation, while none of transconjugants carried *bla*_SHV_, *bla*_GES,_ and AmpC group genes. In addition, one of the PMQR genes (*qnrB*) and aminoglycoside resistance genes (*aac (6′)-Ib, ant (2′)-Ia*) were also successfully transferred along with *β*-lactamase genes (Table 5). Among the 18 transconjugants, all CRKP donors were able to transfer their *bla*_NDM-1_ to the recipient strains, and 87.5% (14/16) were able to transfer *bla*_KPC-2_. Compared to the original donor, the majority of the transconjugants had significantly lower MIC values to carbapenems (IMP), and two transconjugants were sensitive to imipenem, with MICs ≤1 *μ*g/ml. The distribution of MICs of imipenem is shown in Figure 3. Carbapenemase genes were successfully transferred in all of ST307, ST15, ST625, ST37, ST290, ST896, and ST2059 CRKP isolates. The conjugation rate of ST323 strains was 75% (3/4), while only 9.1% (2/22) carbapenemase genes of ST11 isolates were successfully transferred.

Plasmid replicon typing showed that all strains only harbored group *F* (IncF) plasmid. Overall, 17.8% (8/45) of the isolates carried three incompatibility group *F* plasmids (IncFII, IncFIB, and IncFIIK), which all belonged to ST11. Among the 45 CRKP isolates, the most common plasmids were IncFIB (66.7%, 30/45), IncFII (66.7%, 30/45) and IncFIIK (37.8%, 17/45). IncFIA, IncHI1, IncI1, IncX, IncA/C, IncL/M, IncB/O, and CoIE plasmids were not detected in our study. The distribution of plasmids among all CRKP is mentioned in Figure 1.

## 4. Discussion

With the use of antibiotics, CRKP has gained extensive attention globally [22]. While previous studies have seldom focused on the molecular epidemiological data of CRKP infections in Northern China. Therefore, we aim to explore the epidemiological characteristics of CRKP isolates in Northern China and to elucidate their drug resistance mechanisms, so as to guide the clinicians to empirically use of antibiotics.

In this study, we investigated the antimicrobial resistance profiles, molecular typing, antibiotic resistance genes and plasmid replicon types of 45 consecutively collected CRKP isolates in Northern China. From the clinical department distribution, most of the CRKP isolates (48.9%, 22/45) originated from the ICU department and showed multidrug resistance, which was consistent with previous studies [23–25]. As *K*. *pneumoniae* is an opportunistic pathogen, ICU patients with impaired immunity are at an increased risk of infection. In particular, some ICU patients also required mechanical ventilation and other invasive operation, so they prone to suffer from a nosocomial infection.

The antimicrobial resistance test showed that they had higher resistance rates to carbapenems, cephalosporins, and fluoroquinolone and lower resistance rates to amikacin, tigecycline, and colistin, which was consistent with CHINET data in 2018 [26]. Because carbapenemase genes and ESBL genes often coexisted in CRKP isolates, the resistance rate of CRKP isolates to cephalosporins was also high, while amikacin, tigecycline and colistin were rarely used in clinical practice, so the resistance rates to these antibiotics were low. It was reported that combination therapy was related to a lower mortality rate than monotherapy in CRKP, and imipenem combined with polymyxin and tigecycline showed high synergistic antibacterial effects in in vitro drug susceptibility tests [27].

All CRKP isolates were found to produce carbapenemases, one of the major mechanisms for carbapenem resistance in CRE. Our results showed that 88.9% isolates produced serine carbapenemase, and 11.1% isolates produced both serine carbapenemase and metactamallo-*β*-lases. Among serine carbapenemases, KPC-2 was the predominant type, which accounted for 93.3% of the isolates, followed by GES (37.8%, 17/45). These results were consistent with the previous studies that found KPC was the most common carbapenemase in China, while GES was higher and NDM was lower compared with other regions in China [28]. The above data indicated that the distribution of carbapenemase genes in CRKP may be varied in different regions. The clinician may use ceftazidime-avibactam as the first line drugs for CRKP infections in Northern China. Most of the CRKP isolates (60%, 27/45) harbored only one carbapenemases gene, 16 isolates carried two carbapenemases genes; with KPC-2 + GES the most frequent type and 2 isolates harbored 3 carbapenemases genes. KPC-2 + GES producing CRKP had a higher MIC_50_ and MIC_90_ compared to KPC-2 producing CRKP.

MLST is a nucleotide sequence-based method that is adequate for characterizing the genetic relationships among bacterial isolates [29, 30]. The most common clinical CRKP strains are those of the clonal group 258 (CG258), which was known to spread throughout the world [31]. Among which, ST258 and ST11 were the most prevalent STs in different parts of the world [32]. ST258 has contributed significantly to the dissemination of carbapenem resistance and has become particularly prevalent in the United States, Latin America, and several European countries [33, 34], while in Asia, the dominant clone is ST11 CRKP, which accounts for up to 60% of CRKP in China [35]. Combined with precise epidemiological information and the characterization of antibiotic resistance mechanisms, MLST analysis of larger sample sets should provide a much improved understanding of the evolutionary origin and dissemination of *K. pneumoniae* MDR strains. In our research, ST11 is still the most common sequence type, accounting for 47.8% (22/45) CRKP isolates, which is consistent with the fact that ST11 is the primary sequence type in Asia for CRKP [36]. While no ST258 CRKP was isolated in our study. In addition, KPC-2 and GES co-producing (14/22) was the main mechanism for carbapenems resistance in ST11 isolates, and national surveillance study on carbapenem nonsusceptible *K*. *pneumoniae* found that the clonal spread of ST11 KPC-2-producing *K. pneumoniae* was occurring at an alarming speed [37, 38]. No NDM-1 or IMP producing ST11 strains were detected. Only one ST2059 isolate was found to produce IMP-type carbapenemase and co-produced NDM-1-type carbapenemase, moreover, NDM-1-type carbapenemase was also detected in ST307, ST625, and ST1 isolates. Among 45 CRKP isolates, the second most common sequence types were ST307 (8.9%, 4/45) and ST323 (8.9%, 4/45). The clonal spread of ST307 was consistent with previous reports, whereas the clonal spread of ST323 increased in our research [39, 40]. Meanwhile, ESBL-producing isolates tend to be multidrug-resistant, and they have an increased risk of treatment failure [41, 42]. Hence, it is apparent to monitor the molecular epidemiology profiles of ESBL-producing isolates in our country. Based on the report by Ocampo et al. all ESBL-producing isolates produced the SHV ESBL enzyme, which was in line with our results [43]. For the CTX-M group, CTX-M-1 was the most common type, and CTX-M-15 was closely related to ST1 [44]. However, CTX-M-2 was the primary CTX-M-type (44/45, 97.8%) and CTX-M-15 was associated with ST307, ST15, and ST2059 in our study.

Plasmids have the ability to carry multiple antibiotic resistance genes (ARGs) and can transfer between the same or different species by conjugation, which makes them play an important role in the dissemination of antimicrobial resistance [45]. In our research, a total of 11 types of plasmid replicons were detected by PCR-based replicon typing method, but only group *F* (IncF) plasmids were identified among the 45 CRKP isolates. Eight isolates (8/45, 17.8%) all belonged to the ST11 clone co-harbored IncFII, IncFIB, and IncFIIK plasmid replicons. IncF-group plasmids were the most common plasmid types, and most of them were associated with ESBLs and carbapenemases, so they were related to the spread of determinants of antimicrobial resistance in *Enterobacteriaceae,* and the surveillance of antimicrobial resistance should be strengthened [46]. It has also been reported that the IncFIB plasmid replicons were closely related to the majority of the antimicrobial resistance genes [47]. As we know, the IncX plasmid was an important vehicle with high mobility in the worldwide dissemination of NDM-1-type carbapenemase [48], while the IncX plasmid was not found and *bla*_NDM-1_ was less common in our study.

There are also some limitations to our study. We only collected CRKP isolates in one large tertiary hospital in Northern China. Multi-center isolates should be collected to provide more evidence on the clinical significance of these resistance strains.

## 5. Conclusions

In conclusion, ICU is the main ward for rapid and widespread transmission of CRKP in a tertiary hospital in Northern China. ST11 is the most frequently cloned and KPC-2 is the main genotype. KPC-2-ST11 is representative clonal lineages. Therefore, knowledge of the molecular epidemiology characteristics and drug-resistant mechanisms of CRKP is crucial in preventing the occurrence and rapid spread of nosocomial infections in tertiary hospitals in Northern China.

## Figures and Tables

**Table 1:** Clinical and demographic characteristics of CRKP patients.

Characteristics	*n* = 45
Median age (years)	53
*Category by age (years)*, *n* (%)	
<20	3 (6.7%)
20–40	6 (13.3%)
41–60	23 (51.1%)
61–80	10 (22.2%)
81–100	3 (6.7%)
Male gender	27 (60.0%)
*Specimen*	
Sputum	18 (40.0%)
Blood	16 (35.6%)
Pus	6 (13.3%)
Ascites	2 (4.4%)
Cerebrospinal fluid	1 (2.2%)
Bile	1 (2.2%)
Secretions	1 (2.2%)
*Wards*	
ICU	22 (48.9%)
Hepatobiliary surgery	10 (22.2%)
Neurosurgery	5 (11.1%)
Hematology	5 (11.1%)
Other departments	3 (6.7%)
*Diseases*	
Brain disease	14 (31.1%)
Pancreatic disease	7 (15.6%)
Blood disease	5 (11.1%)
Biliary disease	5 (11.1%)
Abdominal pain	5 (11.1%)
Heart disease	3 (6.7%)
Hepatopathy	3 (6.7%)
Other diseases	3 (6.7%)

ICU, intensive care unit; CRKP, carbapenem-resistant*Klebsiella pneumoniae*.

**Table 2:** The antimicrobial resistance profiles of 45 CRKP isolates.

Antibiotic	CRKP (*n* = 45)	ST11 (*n* = 22)	Others-ST (*n* = 23)	*P* value	ICU (*n* = 22)	Non-ICU (*n* = 23)	*P* value
	Total resistance rate (%)	Resistance rate (%)	Resistance rate (%)		Resistance rate (%)	Resistance rate (%)	
TZP	77.80	68.20	87.00	0.001	72.70	82.60	0.088
AMC	100.00	100.00	100.00	—	100.00	100.00	—
SCF	100.00	100.00	100.00	—	100.00	100.00	—
CFZ	100.00	100.00	100.00	—	100.00	100.00	—
CXM	100.00	100.00	100.00	—	100.00	100.00	—
CRO	100.00	100.00	100.00	—	100.00	100.00	—
FEP	77.80	72.70	82.60	0.088	72.70	82.60	0.088
FOX	71.10	63.60	78.30	0.029	63.60	78.30	0.029
IPM	100.00	100.00	100.00	—	100.00	100.00	—
MEM	100.00	100.00	100.00	—	100.00	100.00	—
ETP	100.00	100.00	100.00	—	100.00	100.00	—
GEN	40.00	31.80	47.80	0.021	40.90	39.10	0.773
AMK	22.20	31.80	13.00	0.001	27.30	17.40	0.088
ATM	93.30	100.00	87.00	<0.001	100.00	87.00	<0.001
TOB	35.60	31.80	39.10	0.301	40.90	30.40	0.104
CIP	91.10	100.00	82.60	<0.001	100.00	82.60	<0.001
LEV	77.80	100.00	56.50	<0.001	90.90	65.20	<0.001
SXT	60.00	50.00	69.60	0.004	63.60	56.50	0.273
TGC	6.70	4.50	8.70	0.268	4.50	8.70	0.268
COL	11.10	9.10	13.00	0.366	4.50	17.40	0.007

TZP, piperacillin/tazobactam; AMC, amoxicillin/clavulanic acid; SCF, cefoperazone-sulbactam; CFZ, cefazolin; CXM, cefuroxime; CRO, ceftriaxone; FEP, cefepime; FOX, cefoxitin; IPM, imipenem; MEM, meropenem; ETP, ertapenem; GEN, gentamicin; AMK, amikacin; ATM, aztreonam; TOB, tobramycin; CIP, ciprofloxacin; LEV, levofloxacin; SXT, trimethoprim/sulfamethoxazole; TGC, tigecycline; COL, colistin.

**Table 3:** Imipenem MICs of different STs of 45 CRKP isolates.

Resistance profile	*N* (%)	No. of isolates by MIC (*μ*g/mL)							MIC_50_ (*μ*g/mL)	MIC_90_ (*μ*g/mL)
		4	8	16	32	64	128	256		
ST11	22 (48.9)	8	3	3	5	2	0	1	8	64
ST307	4 (8.9)	3	0	0	0	1	0	0	4	64
ST323	4 (8.9)	1	3	0	0	0	0	0	8	8
ST76	3 (6.7)	0	2	1	0	0	0	0	8	16
ST15	2 (4.4)	1	1	0	0	0	0	0	4	8
ST625	2 (4.4)	0	0	0	2	0	0	0	32	32
Others-ST	8 (17.8)	4	2	0	1	0	0	1	4	256

ST, sequence type; MIC, the minimal inhibitory concentration; *μ*g/ml, micrograms per milliliter; MIC_50_, minimum inhibitory concentration for 50% of the isolates; MIC_90_, minimum inhibitory concentration for 90% of the isolates.

**Table 4:** Imipenem MICs of 45 CRKP isolates with different carbapenemases, ESBLs, and AmpC combination profiles.

Resistance profile	*N* (%)	No. of isolates by MIC (*μ*g/mL)							MIC_50_ (*μ*g/mL)	MIC_90_ (*μ*g/mL)
		4	8	16	32	64	128	256		
Carb + ESBL + AmpC+	3 (6.7)	0	0	0	3	0	0	0	32	32
Carb + ESBL + AmpC−	42 (93.3)	17	11	4	5	3	0	2	8	64
*Carbapenemase genes*										
KPC-2	25 (55.6)	13	7	1	2	1	0	1	4	32
KPC-2, GES	14 (31.1)	3	3	3	2	2	0	1	16	64
GES	1 (2.2)	0	0	0	1	0	0	0	32	32
NDM-1	1 (2.2)	0	0	0	1	0	0	0	32	32
KPC-2, NDM-1	1 (2.2)	1	0	0	0	0	0	0	4	4
KPC-2, NDM-1, GES	1 (2.2)	0	0	0	1	0	0	0	32	32
KPC-2, NDM-1, IMP	1 (2.2)	0	1	0	0	0	0	0	8	8
GES, NDM-1	1 (2.2)	0	0	0	1	0	0	0	32	32

ESBL, extended-spectrum*β*-lactamase; Carb: carbapenemase; AmpC: AmpC-*β*-lactamase; MIC, the minimal inhibitory concentration; *μ*g/ml, micrograms per milliliter; MIC_50_, minimum inhibitory concentration for 50% of the isolates; MIC_90_, minimum inhibitory concentration for 90% of the isolates.

**Table 5:** Resistance features of the CRKP and transconjugants.

Strains	Genotypes	Plasmid replicons	MIC (*μ*g/ml)									
			IPM	FEP	CRO	FOX	ATM	GEN	AMK	CIP	LEV	TGC
*K*. *pneumoniae*												
**CRKP 3**	*bla *_KPC-2_, *bla*_SHV-11_, *bla*_TEM-1_, *bla*_CTX-M-1_, *bla*_CTX-M-2_, *bla*_CTX-M-3_, *bla*_CTX-M-15_, *bla*_OXA-1_, *acc (6′)-Ib, qepA, oqxA,* and *qnrB*	FIB	4	>16	>32	32	>64	≤1	≤2	>4	>8	2
**CRKP 4**	*bla *_KPC-2_, *bla*_SHV-12_, *bla*_CTX-M-2_, *bla*_CTX-M-9_, *ant(3′)-I, qepA, oqxA*, and *qnrS*	FIB	4	>16	16	8	>64	>16	≤2	>4	4	1
**CRKP 9**	*bla *_KPC-2_, *bla*_SHV-12_, *bla*_CTX-M-2_, *bla*_CTX-M-3_, *bla*_CTX-M-9_, *qepA, oqxA*, and *qnrB*	FII_K_ FIB	256	>16	>32	>64	>64	≤1	≤2	>4	>8	2
**CRKP 12**	*bla *_KPC-2_, *bla*_SHV-12_, *bla*_CTX-M-3_, *bla*_CTX-M-2_, *ant (3′)-I, qepA* and *oqxA*	FII_K_	8	>16	8	>64	16	≤1	≤2	0.5	1	1
**CRKP 15**	*bla *_KPC-2_, *bla*_SHV-11_, *bla*_TEM-1_, *bla*_CTX-M-1_, *bla*_CTX-M-2_, *bla*_CTX-M-3_, *qepA, oqxA,* and *qnrB*	FII	16	>16	>32	>64	>64	≤1	≤2	0.5	1	≤0.5
**CRKP 17**	*bla *_KPC-2_, *bla*_SHV-12_, *bla*_CTX-M-2_, *bla*_CTX-M-3_, *acc (6′)-Ib, acc(3′)-IV, qepA* and *oqxA*	FII FII_K_	8	2	>32	8	>64	>16	≤2	>4	>8	2
**CRKP 18**	*bla *_KPC-2_, *bla*_GES_, *bla*_SHV-33_, *bla*_CTX-M-2_, *bla*_CTX-M-9_, *ant (3′)-I, qepA* and *oqxA*	FIB	32	>16	>32	>64	>64	≤1	≤2	>4	>8	2
**CRKP 32**	*bla *_NDM-1_, *bla*_CTX-M-2_, *bla*_CTX-M-3_, *bla*_OXA-1_, *bla*_DHA_, *acc (6′)-Ib, acc (3′)-IV, ant(3′)-I qepA and qnrB*		16	>16	>32	>64	≤1	>16	>64	>4	>8	4
**CRKP 33**	*bla *_NDM-1_, *bla*_GES_, *bla*_CTX-M-2_, *bla*_CTX-M-3_, *bla*_OXA-1_, *bla*_DHA_, *acc(6′)-Ib, acc(3′)-IV, ant (3′)-I, qepA and qnrB*		16	>16	>32	>64	≤1	>16	>64	>4	>8	4
**CRKP 40**	*bla *_KPC-2_, *bla*_SHV-12_, *bla*_CTX-M-2_, *bla*_CTX-M-3_, *ant (3′)-I, qepA*, and *oqxA*	FII FII_K_	4	>16	>32	32	>64	>16	≤2	1	≤1	≤0.5
**CRKP 41**	*bla *_KPC-2_, *bla*_SHV-11_, *bla*_TEM-1_, *bla*_CTX-M-1_, *bla*_CTX-M-2_, *bla*_CTX-M-3_, *bla*_CTX-M-15_, *bla*_OXA-1_, *acc (6′)-Ib, ant (3′)-IV, ant (3′)-I*, *qepA*, and *oqxA*	FII FII_K_	4	4	>32	>64	>64	>16	4	>4	>8	2
**CRKP 47**	*bla *_KPC-2_, *bla*_SHV-11_, *bla*_TEM-1_, *bla*_CTX-M-2_, *bla*_CTX-M-3_, *bla*_OXA-1_, *acc (6′)-Ib qepA oqxA*, and *qnrB*	FII FIB	4	>16	>32	>64	>64	≤1	≤2	>4	>8	4
**CRKP 63**	*bla *_KPC-2_, *bla*_SHV-12_, *bla*_TEM-1_, *bla*_CTX-M-2_, *acc (3′)-II aac (6′)-Ib, qepA*, and *oqxA*	FII FII_K_ FIB	8	>16	32	>64	>64	>16	≤2	≤0.25	≤1	1
**CRKP 74**	*bla *_KPC-2_, *bla*_SHV-12_, *bla*_CTX-M-2_, *bla*_CTX-M-9_ acc (3′)-*IIa ant(2′)-Ia, qepA, oqxA*, and *qnrS*	FII FII_K_ FIB	4	16	>32	16	>64	>16	≤2	1	≤1	1
**CRKP 82**	*bla *_KPC-2_, *bla*_SHV-11_, *bla*_TEM-1_, *bla*_CTX-M-1_, *bla*_CTX-M-2_, *bla*_CTX-M-3_, *bla*_CTX-M-15_, *bla*_OXA-1_, *acc (6′)-Ib, qepA, oqxA*, and *qnrB*	FII FIB	4	16	>32	8	>64	≤1	≤2	>4	>8	2
**CRKP 85**	*bla *_KPC-2_, *bla*_SHV-12_, *bla*_CTX-M-2_, *bla*_CTX-M-3_, *ant (3′)-I, qepA*, and *oqxA*	FII_K_	8	4	32	32	>64	≤1	≤2	1	≤1	≤0.5
**CRKP 88**	*bla *_KPC-2_, *bla*_NDM-1_, *bla*_SHV-11_, *bla*_TEM-1_, *bla*_CTX-M-1_, *bla*_CTX-M-2_, *bla*_CTX-M-3_, *bla*_CTX-M-15_, *bla*_OXA-1_, *acc (6′)-Ib, qepA, oqxA*, and *qnrB*	FII FIB	4	>16	>32	32	>64	≤1	≤2	>4	>8	2
**CRKP 89**	*bla *_KPC-2_, *bla*_NDM-1_, *bla*_IMP_, *bla*_CTX-M-1_, *bla*_CTX-M-2_, *bla*_CTX-M-3_, *bla*_CTX-M-15_, *ant (3′)-I, qepA,* and *qnrS*		8	>16	>32	>64	≤1	≤1	≤2	1	≤1	≤0.5

*Transconjugants*												
**CRKP 3C**	*bla *_KPC-2_, *acc (6′)-Ib*, and *qnrB*		2	8	8	≤8	>16	≤1	≤2	≤0.5	≤1	2
**CRKP 4C**	*bla *_KPC-2_		4	2	4	≤8	>16	≤1	≤2	≤0.5	≤1	1
**CRKP 9C**	*bla *_KPC-2_, *bla*_CTX-M-9_, and *qnrB*		2	16	>32	≤8	>16	≤1	≤2	≤0.5	≤1	1
**CRKP 12C**	*bla *_KPC-2_	FII_K_	4	16	4	≤8	8	≤1	≤2	≤0.5	≤1	1
**CRKP 15C**	*bla *_KPC-2_ and *qnrB*	FII	4	8	8	≤8	>16	≤1	≤2	≤0.5	≤1	1
**CRKP 17C**	*bla *_KPC-2_ and *acc (6′)-Ib*	FII FII_K_	4	2	8	≤8	>16	≤1	≤2	≤0.5	≤1	2
**CRKP 18C**	*bla *_KPC-2_		2	8	8	≤8	>16	≤1	≤2	≤0.5	≤1	2
**CRKP 32C**	*bla *_NDM-1_, *acc (6′)-Ib* and *qnrB*		8	>16	>32	≤8	≤1	≤1	≤2	≤0.5	≤1	4
**CRKP 33C**	*bla *_NDM-1_, *acc (6′)-Ib*, and *qnrB*		4	>16	>32	≤8	≤1	≤1	≤2	≤0.5	≤1	2
**CRKP 40C**	*bla *_KPC-2_	FII FII_K_	2	8	8	≤8	>16	≤1	≤2	≤0.5	≤1	1
**CRKP 41C**	*bla *_KPC-2_, *bla*_TEM-1_*bla*_OXA-1_*bla*_CTX-M-15_ and *acc (6′)-Ib*	FII FII_K_	4	2	>32	≤8	>16	≤1	≤2	≤0.5	≤1	1
**CRKP 47C**	*bla *_KPC-2_ * acc (6′)-Ib,* and *qnrB*	FII	8	>16	32	≤8	>16	≤1	≤2	≤0.5	≤1	4
**CRKP 63C**	*bla *_KPC-2_ and *acc (6′)-Ib*	FII	4	8	8	≤8	>16	≤1	≤2	≤0.5	≤1	1
**CRKP 74C**	*bla *_KPC-2_ and *ant (2′)-Ia*	FII FII_K_	≤1	8	4	≤8	>16	≤1	≤2	≤0.5	≤1	1
**CRKP 82C**	*bla *_KPC-2_, *acc (6′)-Ib* and *qnrB*	FII	4	8	8	≤8	>16	≤1	≤2	≤0.5	≤1	1
**CRKP 85C**	*bla *_KPC-2_		8	2	16	≤8	>16	≤1	≤2	≤0.5	≤1	1
**CRKP 88C**	*bla *_NDM-1_, *acc (6′)-Ib* and *qnrB*	FII	≤1	>16	8	≤8	>16	≤1	≤2	≤0.5	≤1	1
**CRKP 89C**	*bla *_NDM-1_		8	>16	>32	≤8	≤1	≤1	≤2	1	≤1	2

IPM, imipenem; FEP, cefepime; CRO, ceftriaxone; FOX, cefoxitin; ATM, aztreonam; GEN, gentamicin; AMK, amikacin; CIP, ciprofloxacin; LEV, levofloxacin; TGC, tigecycline.

## Data Availability

The data are obtainable from the corresponding authors upon reasonable request.
